# Left Pubic Symphysis Fracture With Undiagnosed Ipsilateral Femoral Neck Fracture in an Elderly Female With Osteoporosis: A Case Report

**DOI:** 10.7759/cureus.69203

**Published:** 2024-09-11

**Authors:** Blake E Delgadillo, Jason S DeFrancisis, Luke Henwood, Justin R Federico

**Affiliations:** 1 Orthopedic Surgery, Lake Erie College of Osteopathic Medicine, Bradenton, USA; 2 Internal Medicine, Baptist Health, Jacksonville, USA

**Keywords:** undiagnosed fracture, hemiarthroplasty, total hip arthroplasty, radiographic imaging, orthopedic injury, computed tomography, x-ray, elderly patients, osteoporosis, femoral neck fractures

## Abstract

Femoral neck fractures are a common complication of falls, particularly in the osteoporotic elderly female population. This case highlights the significance of properly radiologically evaluating elderly patients with falls. A 68-year-old White female with paroxysmal atrial fibrillation on anticoagulation, nicotine use disorder, chronic hyponatremia, hypertension, and hyperlipidemia presented to the emergency department (ED) in considerable pain four days after a previously diagnosed left pubic symphysis fracture. Repeat imaging (X-ray and computed tomography (CT)) was significant for a left femoral neck fracture concurrent with the aforementioned pelvic fracture. Within 48 hours of admission, the patient underwent a hemiarthroplasty of the left hip. The postoperative course was unremarkable, except for immensely decreased pain and a remarkable ability to ambulate, and on postoperative day three, the patient was discharged. In patients with an overall high risk of hip fracture, CT imaging should be considered after X-ray in an effort to eliminate the frequency of undiagnosed fractures. It is essential to thoroughly evaluate the entire film, even after one fracture is found. This is crucial to a patient’s overall well-being and can contribute to many unwanted phenomena.

## Introduction

Hip fractures are a common injury, particularly in the elderly population. There are approximately 1.6 million hip fractures that occur annually worldwide [1]. In the United States, there are an estimated 150,000 hip fractures yearly, and it is estimated that there will be 300,000 hip fractures annually by the year 2030 [2,3]. The estimated costs of hip fracture care are between $10.3 billion and $15.2 billion [2].

The incidence of hip fractures increases with patient age, with an average age of 80 years [4]. Femoral neck fractures, a type of intracapsular hip fracture, are the most common type of hip fracture. They are often associated with low-energy falls in the elderly and are more commonly seen in female patients [5]. The risk of hip fracture increases in patients with several comorbidities, such as nicotine use disorder and osteoporosis. Out of all osteoporotic fractures, hip fractures make up 13.7% [6,7]. Additional factors such as frequency of falls, low bone mass, low body weight, low physical activity, estrogen deficiency, and earlier fracture also increase the risk of hip fractures [8]. Complications that can arise with femoral neck fracture include disrupting the blood supply from the medial femoral circumflex artery to the femoral head, which can disrupt the healing process and even lead to avascular necrosis [9].

Hip fractures often present with pain, inability to walk, and a classic deformity of a shortened and externally rotated limb [4]. The majority of hip fracture cases are diagnosed with X-ray, which is the standard initial imaging after trauma [10]. In 4-9% of cases in which patients present with pain after trauma, these fractures can go undiagnosed due to a variety of reasons, which may include the level of expertise of the interpreter, patient age, perception errors, and insensitive imaging methods [11]. Fractures that are undiagnosed and receive delayed treatment are associated with greater rates of postoperative complications and a higher 30-day mortality rate [12]. In the treatment of a femoral neck fracture, depending on the fracture type and location, conservative and operative options are available, including closed or open reduction and internal fixation, in situ fixation via dynamic hip screws or sliding hip screws, hemiarthroplasty, and total hip arthroplasty [13]. This case highlights the significance of properly radiologically evaluating potential hip fractures in high-risk elderly patients after falls and underscores the vitality of clinicians diagnostically interpreting a patient’s imaging in addition to fully evaluating the radiology report.

## Case presentation

**Figure 1:**
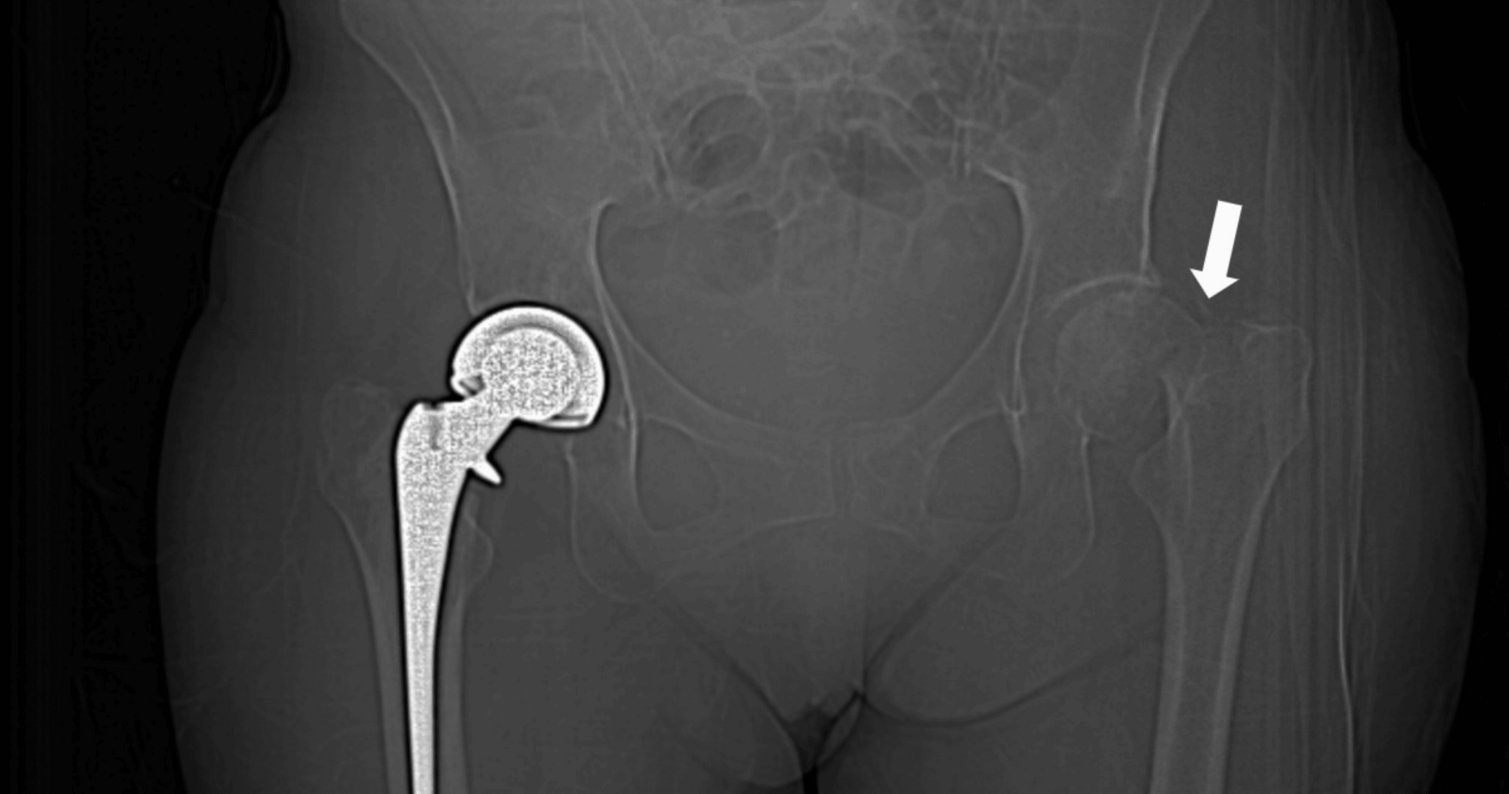
Anteroposterior radiograph (scout computed tomography) of the pelvis showing a left femoral neck fracture Anteroposterior radiograph (scout computed tomography) of the pelvis demonstrating a non-displaced fracture of the left pubic symphysis. There is also a left subcapital femoral neck with shortening, possible comminution, lateral displacement, and varus angulation. A previous right total hip arthroplasty is appreciated.

**Figure 2:**
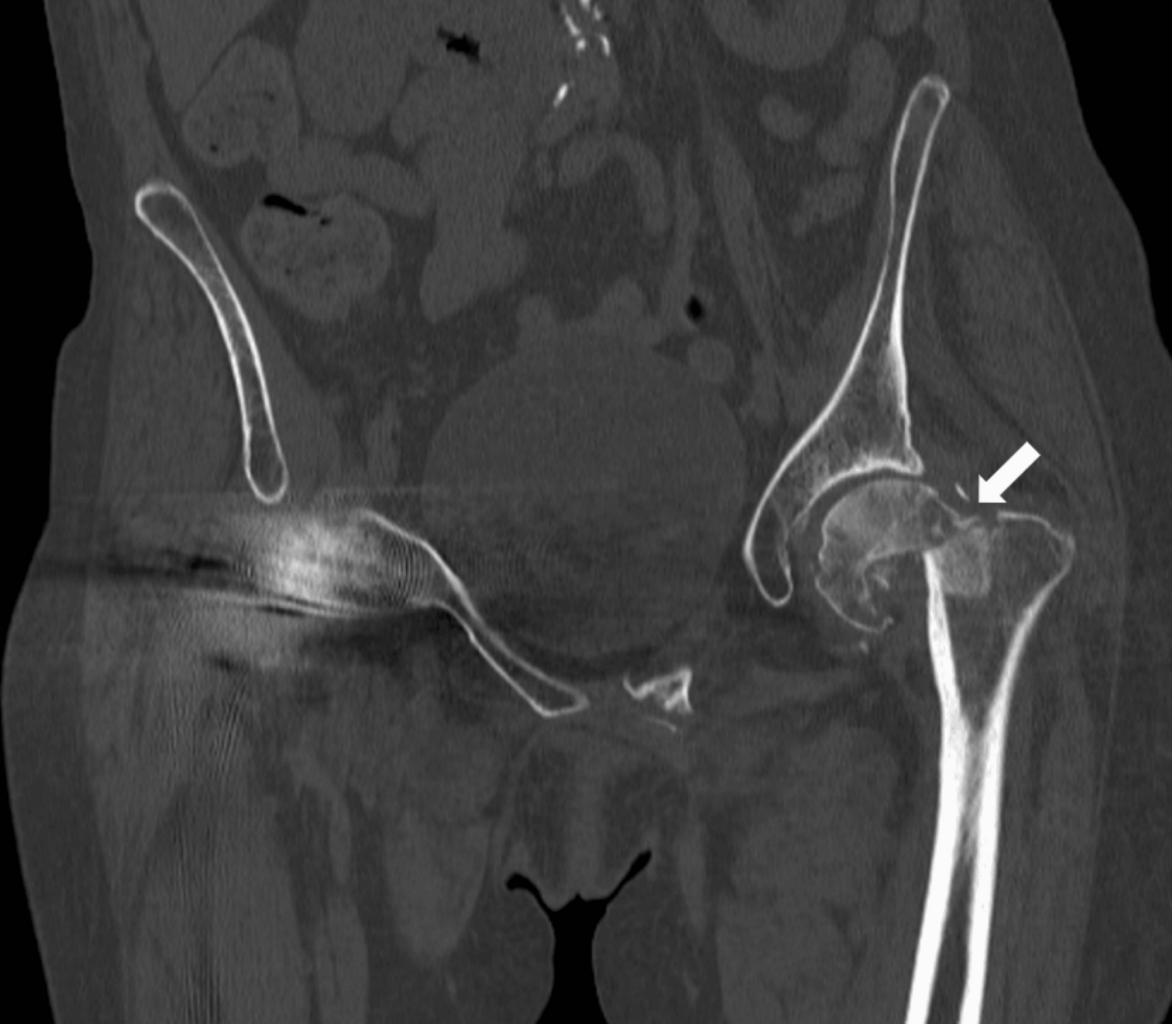
Coronal computed tomography of the pelvis showing a left femoral neck fracture Coronal computed tomography of the pelvis displaying a left subcapital femoral neck with shortening, minimal comminution, lateral displacement, and varus angulation.

**Figure 3:**
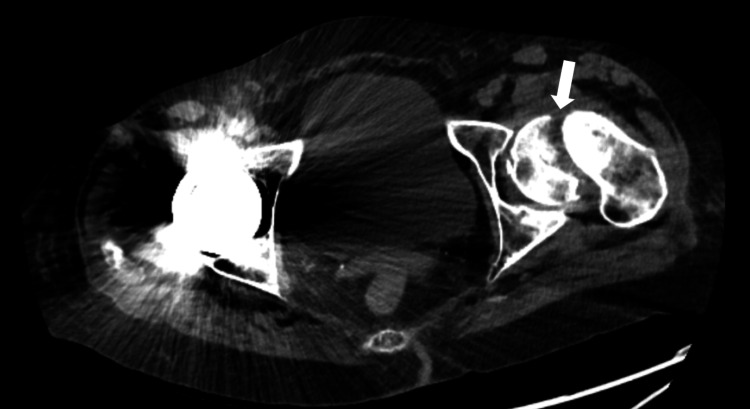
Transverse computed tomography of the pelvis showing a left femoral neck fracture Transverse computed tomography of the pelvis exhibiting a left subcapital femoral neck fracture with anterior angulation, slight impaction, and small comminution. A previous right total hip arthroplasty and resulting artifact are present.

**Figure 4:**
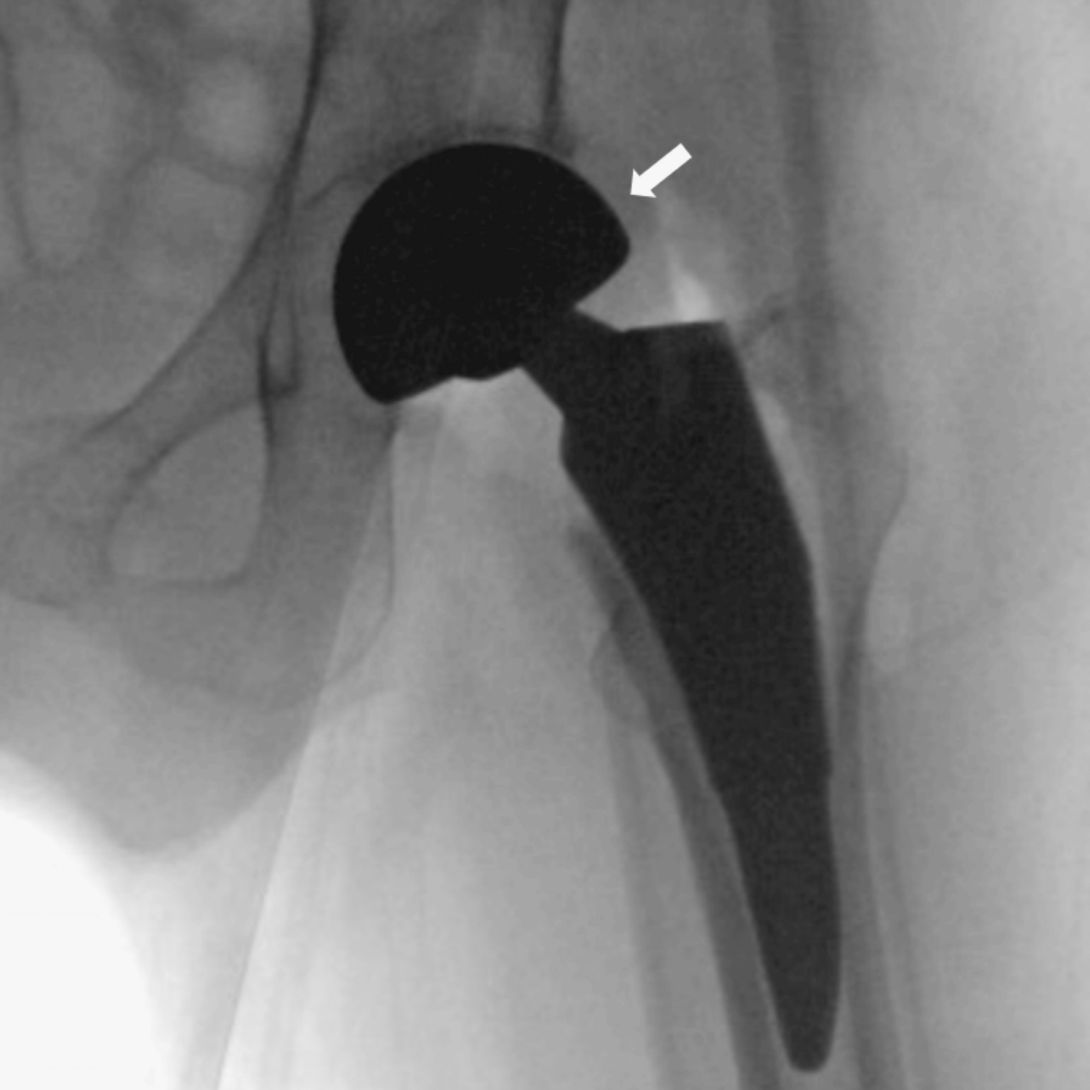
Intraoperative anteroposterior radiograph of left hip showing hemiarthroplasty prosthesis An intraoperative anteroposterior radiograph of the left hip showing a hemiarthroplasty prosthesis in the proper, expected position. There is no appreciable fracture or dislocation.

**Figure 5:**
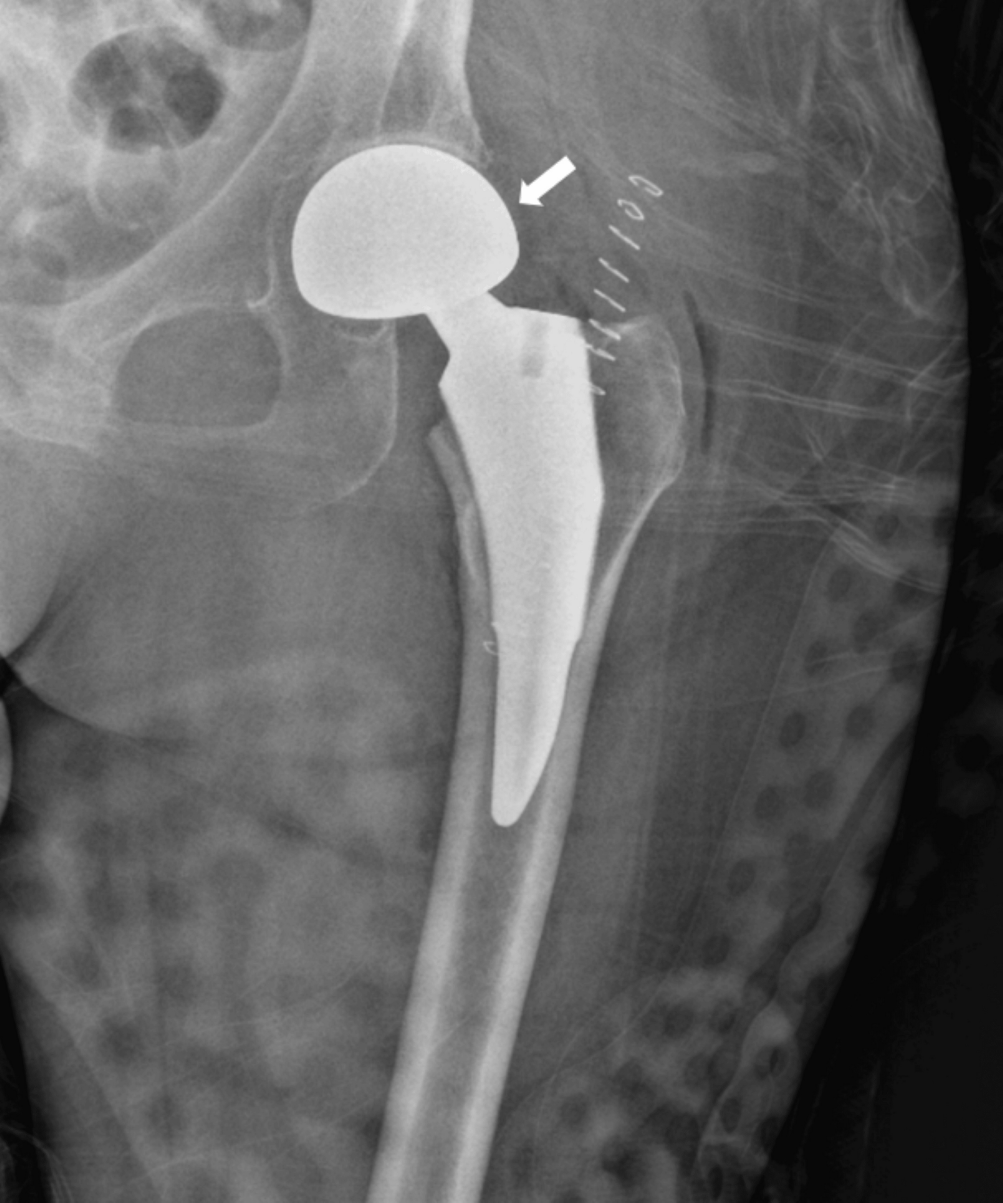
Anteroposterior radiograph of the left hip status post left hip hemiarthroplasty Anteroposterior radiograph of the left hip status post left hip hemiarthroplasty showing a left hip prosthesis in proper alignment with skin staples in place. No fracture or dislocation is appreciated.

A 68-year-old White female with paroxysmal atrial fibrillation on anticoagulation, nicotine use disorder, chronic hyponatremia, hypertension, and hyperlipidemia presented to the emergency department (ED) in considerable pain four days after a previously diagnosed (via X-ray) left pubic symphysis fracture subsequent to a ground level fall. In the ED, repeat imaging with X-ray and computed tomography (CT) was significant for a left femoral neck fracture concurrent with the aforementioned pelvic fracture. Mild joint space narrowing was also noted (Figures 1-3). Within 48 hours of admission, the patient underwent a hemiarthroplasty of the left hip, and intraoperative and postoperative radiographs showed a well-positioned hemiarthroplasty prosthesis (Figures 4, 5). The postoperative course was unremarkable, except for a mild leukocytosis that was resolved with antibiotics. She reported immensely decreased pain and a remarkable ability to ambulate, and on postoperative day three, the patient was discharged. Upon outpatient follow-up, the patient reported that she was doing well with her quality of life. She was instructed to return to her primary care physician for osteoporosis management.

## Discussion

In patients at high risk of hip fracture, including elderly females with a past medical history of osteoporosis, CT imaging should be considered after X-ray in the acute setting. Although X-ray is the initial imaging of choice for the detection of hip fractures after trauma, it offers only a moderate diagnostic accuracy, which puts patients at higher risk of undiagnosed fractures [10]. In addition to CT imaging, clinicians should be encouraged to interpret a patient’s imaging in addition to fully evaluating the radiology report, even in cases of common diagnoses. In general, there have been reports of up to 3-5% of radiology studies containing errors and discrepancies in their reports [14]. Of the estimated 150,000 hip fractures that occur annually, the one-year mortality rate is reported to be between 18% and 31% [2,4]. Adding multiple layers to evaluating a patient’s condition may have the potential to decrease the amount of missed fractures.

In particular, femoral neck fractures are largely treated operatively, as surgical intervention is typically indicated to control pain, preserve mobility, and stimulate functional recovery and osseous healing [15]. Surgical intervention should not be delayed; therefore, clinicians need to identify fractures properly, as delays in surgical intervention lead to increased mortality rates [12]. Clinical judgment and the Garden classification should be used in selecting a surgical technique for femoral neck fractures [16]. Femoral neck fractures that are displaced are classified as Garden type III or IV and treated with hemiarthroplasty or total hip arthroplasty [16].

In elderly patients with minimal activity levels and displaced intracapsular femoral neck fractures, hemiarthroplasty is indicated [15]. In elderly patients with high levels of activity possessing intact ambulatory function or evidence of hip pain and degenerative arthritis, total hip arthroplasty is indicated [15]. In patients younger than 80 years old and elderly patients with a life expectancy greater than four years post-operation, total hip arthroplasty is recommended for displaced femoral neck fractures [17]. Non-operative management is rarely indicated in femoral neck fractures; it is used for non-ambulatory patients with significant medical comorbidities [15].

Careful image reading by clinicians on top of a radiologist report can help ensure that patients are treated appropriately and in a timely manner. When fractures fail to be properly treated, they can lead to chronic pain, mechanical compensation, muscular imbalance, internal bleeding, and potential avascular necrosis [18]. In particular, delaying treatment of hip fractures in the elderly is associated with increased 30-day mortality and rates of postoperative complications [12]. In addition, the estimated one-year mortality has been shown to increase up to 26.8% in patients over 65 years of age following a femoral head or neck fracture [19]. Functional recovery in the elderly with hip fractures has been noted to be less than 50%, and approximately 25% of elderly patients with these fractures result in greater than one year of stays in long-term care facilities [6].

In addition to the necessity of proper identification on imaging and careful rereading of images, clinicians should educate high-risk patients on the classic presentation of hip fractures. Among elderly patients, there is poor awareness of the clinical presentation of hip fractures, which can lead to delays in patients seeking treatment and further complications [5].

A factor that can hinder clinicians’ ability to read a patient’s imaging is the limited amount of time physicians have per patient. On average, an orthopedic surgeon sees 31 patients daily [20]. The high volume of patients per day may limit the time spent with each patient. Despite the limited available time, clinicians should strive to read all their images in pursuit of alleviating undiagnosed fractures.

## Conclusions

The case at hand should serve as an example for clinicians with a high suspicion of undiagnosed fractures to further radiologically evaluate their patients, even in the outpatient setting. Complementary to reading written X-ray reports, clinicians should be encouraged and guided to interpret a patient’s X-ray imaging before ordering CT imaging, which may help reduce missed fractures. Additionally, CT imaging may help decrease the rate of undiagnosed fractures on initial X-rays. This guidance is especially vital for patients with multiple risk factors, including osteoporosis, elderly age, and female gender, where suspicion should be further increased. The utilization of supplementary radiological imaging may help prevent the continuation of pain, further destruction of native anatomy, worsening fracture alignment, and mortality.
